# A New Model for Secondary Prevention of Stroke: Transition Coaching for Stroke

**DOI:** 10.3389/fneur.2014.00219

**Published:** 2014-10-27

**Authors:** Cheryl Bushnell, Martinson Arnan, Sangwon Han

**Affiliations:** ^1^Department of Neurology, Wake Forest School of Medicine, Winston Salem, NC, USA; ^2^Department of Neurology, Sanggye Paik Hospital, Inje University College of Medicine, Seoul, South Korea

**Keywords:** stroke secondary prevention, medication adherence, quality improvement

## Abstract

Non-adherence to stroke prevention medications is a risk factor for first-ever and recurrent stroke. As of yet, there are no guidelines for processes to recognize and address medication non-adherence in stroke patients. We developed a new model of post-discharge prevention care that measures and addresses medication-taking (transition coaching for stroke or TRACS). TRACS includes personalized education about risk factors and medications prior to discharge, follow-up telephone calls, and appointments with a stroke nurse practitioner (NP). The stroke NP asks about medication use (persistence) and whether doses are missed (adherence), and helps to solve problems with access to medications or side effects. In an analysis of 142 patients enrolled in TRACS from October 2012 to February 2014, medication persistence (use of medications from discharge to the time of measurement) was about 80%. Medication persistence at NP visit was higher in those patients with a first-ever stroke (78.9%) vs. those with recurrent stroke (60.7%; *p* = 0.045). Concerted efforts with 2-day RN follow-up calls and earlier NP appointments to improve medication-taking behaviors are underway.

## Introduction

There are approximately 795,000 new strokes per year in the US, 23% of which are recurrent (1). The risk factors for stroke are well-recognized, and risk-stratification according to the presence of diabetes, hyperlipidemia, atrial fibrillation, current tobacco smoking, and hypertension occurs primarily during the hospitalization for acute stroke. Medications are then prescribed to modify these risk factors with the intent of reducing the risk of recurrent stroke. Evidenced-based guidelines focused on secondary prevention of stroke provide recommendations for treatments for each of these risk factors (2).

The processes of improving risk reduction during the acute hospital stay have been incorporated into quality improvement programs such as American Heart Association/American Stroke Association’s get-with-the-guidelines – stroke and Paul Coverdell Acute Stroke Registries. These programs provide feedback on processes of care that are now publicly reported as core performance measures, such as measurement of a lipid profile and treatment with a statin, anticoagulation for atrial fibrillation, and antithrombotic therapy for prevention early in the hospital course (hospitalcompare.gov). These programs, however, are not specifically designed to maintain stroke prevention efforts after discharge.

Prescription of evidenced-based stroke prevention medications is only the first step. If patients are not willing or able to take these medications, then the action was futile. The final frontier for improving the quality of stroke secondary prevention, therefore, is finding ways to improve medication adherence (3). A recent Cochrane Systematic Review of randomized controlled trials testing educational and behavioral interventions to improve stroke prevention (including adherence to prescribed medications) was published. Unfortunately, the authors reported that none of these interventions led to significant improvement in medication adherence or recurrent cardiovascular events (4). More work is needed to identify effective strategies to improve adherence and thus enhance the effectiveness of secondary prevention.

### Medication non-adherence: An under-recognized risk factor for stroke

Non-adherence to medications in patients at risk for stroke is an important risk factor in and of itself. For example, the heart and soul study showed that in patients with coronary artery disease, if patients only took 75% or less of their medications as prescribed, the risk for stroke was fourfold higher than patients who were 100% adherent (5). Similarly, a *post hoc* analysis of the vitamins in stroke prevention (VISP) study investigated the impact of general non-adherence to study medication, whether it was the active vitamin preparation or placebo and the impact on the outcome. Compared to <65% adherence, pill adherence levels of ≥90 to <99 and ≥99% were associated with a 44% (*p* = 0.02) and 54% (*p* = 0.001), respectively, lower occurrence of the combined outcome of stroke, myocardial infarction, or death at 18 months (6). A study of Tennessee’s Medicaid program from 1994 to 2000 showed that antihypertensive medication adherence by one pill per week for a once-a-day regimen reduced the risk of stroke by 8–9% and death by 7% (7). A separate analysis of stroke patients in a Medicaid program showed that stroke recurrence was 57% less likely if patients consistently took medications over time (i.e., persistent), even after adjusting for confounders (8). Unfortunately, stroke patients are a high-risk group just by virtue of having suffered one. For example, in the international reduction of atherothrombosis for continued health (REACH) registry, experiencing a non-fatal stroke was a predictor of non-adherence with cardiovascular prevention medications (9). Therefore, overwhelmingly the evidence points to adherence as a critically important factor in stroke prevention, and yet screening for and recognizing non-adherence is not yet an established part of any process measure of quality for stroke.

### What is known about medication adherence in stroke?

Medication-taking behavior can be categorized as persistence or adherence. Medication persistence is defined as the duration of therapy, whereas adherence is the extent to which patients take their medications as prescribed (i.e., not missing doses). The adherence evaluation after ischemic stroke – longitudinal (AVAIL) registry was designed *a priori* to account for the patient, provider, and system factors that may impact medication persistence/adherence in stroke patients (10). AVAIL used self-reported 3-month secondary medication regimen persistence (comparing medication lists at discharge and at 3 and 12 months after stroke) as the primary outcome. The primary findings were that medication persistence at 3 months was 75%. Some of the factors associated with persistence were decreasing number of medications, history of hypertension, diabetes, and hyperlipidemia, less severe stroke disability, higher quality of life, having insurance, working status, and understanding why medications are prescribed and how to refill them (11). At 1 year, non-persistence (discontinuation of a medication for any reason, including provider recommendations) and non-adherence (discontinuation for reasons other than provider recommendations) were analyzed separately. One-year persistence (65.6%) and adherence (86.6%) differed, based on the assumption that provider-recommended discontinuation accounted for most, but not all, of the non-persistence. Some factors associated with persistence and/or adherence included decreasing number of medications, adequate income, lesser disability from stroke, having an appointment with a primary care physician (PCP), using a pillbox, having insurance, receiving medication instructions at discharge, marital status, and being discharged to home (12).

The results from other studies of medication-taking among stroke patients have reported considerable variability. Analysis of a Medicaid managed care database from Maryland reported the persistence in stroke patients was about 80% after maximum follow-up of 2 years (8). In Nova Scotia, Canada, self-reported medication persistence at 6 months ranged from 83.3% for anticoagulants to 97.1% for anti-hyperglycemic drugs. At 12 months, the ranges were nearly identical for the various categories of medications (13).

In contrast, an analysis of over 21,000 stroke survivors in the Sweden showed a steady decline in medication persistence from discharge to 2-year follow-up (14). After 2 years, medication persistence ranged from 74% for antihypertensive drugs to only 45% for warfarin. The factors associated with non-persistence in this cohort were advanced age, comorbidity, good self-perceived health, absence of low mood, acute treatment in a stroke unit, and institutional living at follow-up (14). These studies illustrate the variability in persistence across different settings, which could relate to heterogeneity in healthcare systems, cultural factors, and methods to measure adherence.

### Strategies to improve adherence to stroke prevention medications

The preventing recurrence of thromboembolic events through coordinated treatment (PROTECT) program was a quality improvement program designed to increase adherence with medications and behavioral changes after stroke and acute coronary artery syndrome (15). Patients admitted to a single center were discharged with materials related to appointments, and at 2–4 weeks, a nurse contacted patients by telephone to reinforce medication/behavior regimens. In the 130 patients followed up at 3 months, adherence to antithrombotics was 100%, statins 99%, angiotensin-converting enzyme inhibitors/angiotensin receptor blockers 92%, thiazides 80%, diet 78%, exercise 70%, and smoking cessation 83% (15). At 1 year, persistence in 128 patients was maintained at a similar rate, demonstrating that medications can be successfully improved and maintained through early initiation and encouragement after discharge (16). This study, however, had no control group.

The AVAIL results showed that specific patient-related factors, such as knowledge of why medications are taken and how to refill them were associated with medication persistence at 3 months. Therefore, we designed a medication coaching intervention that would focus on these factors as well as providing non-medication help for patients or families that needed it (17). In a two-arm study of stroke patients who had a change of at least 2 prevention medications between admission and discharge, 20 were assigned to the intervention, and 10 to usual care. The medication coach enrolled patients in the hospital prior to discharge and provided educational materials. She then contacted participants at about 2 weeks after discharge to review medications and to triage open-ended questions about medications to a pharmacist or questions about stroke to a stroke nurse. The information was then compiled and given to the participant by telephone. Overall, the program was well-received by those who received the intervention, and we noted a trend toward improved appointment-keeping with primary care in those who received the coaching. Not surprisingly, the sample was too small to assess differences in medication persistence (17). The theme of the questions asked by patients and caregivers focused on how to prevent another stroke, and how to take medications to avoid interactions. We learned that although many of the questions and concerns about medications and stroke were similar, there were other system failures, such as difficulties obtaining appointments, and social issues, such as lack of transportation to appointments for providers and physical/occupational/speech therapy. Medication adherence is obviously critically important, but as we learned in our coaching intervention, it was actually the tip of the iceberg. An effective prevention model would require recognition of medication non-adherence and also readiness by the team to provide individualized assistance and education during the transition from hospital to home.

## A New Model of Stroke Prevention

### TRACS program development

The medication coaching pilot study informed the development of the transitional coaching for stroke (TRACS) program, a hospital-supported quality improvement program to reduce 30-day readmissions, maximize stroke prevention, and improve outcomes. The TRACS program provides one-on-one transition coaching to patients admitted with an ischemic or hemorrhagic stroke or TIA. The TRACS coach meets individually with patients prior to discharge and provides a take-home packet with a personalized review of the patient’s risk factors, medication information, instructions for stroke awareness, action with new symptoms, and post-hospital follow-up care. These materials are given in large font for a 7–8th grade reading level with a one page summary. The coach explains new medications that have been added to the patient’s regimen, behavioral changes that are necessary for a successful transition and also answers any questions the patient may have. Also, during transition coaching, the TRACS Program Baseline Information Survey is administered, which collects information regarding the health status, health literacy, depression, insurance status, adequacy of income, and premorbid status.

After discharge, the TRACS coach (initially an educator, now an RN) calls the patient within 2 weeks to assess medications, any new problems after arriving home, and confirm appointments with the patient or caregiver if patient is unable or unavailable to speak to the coach. The next contact is in the stroke nurse practitioner (NP) follow-up clinic between 2 and 4 weeks, where a standardized assessment is performed. This includes screening for new stroke symptoms, depression, and fall risk, assessing progress with rehabilitation, providing referrals to home health if needed, confirming access to primary care, and discussion of diagnostic test results from the stroke hospitalization. Importantly, self-reported medication use and reconciliation is obtained at this visit, with follow-up questions related to why a medication is not taken and why, as well as steps to help with access to cheaper alternatives if cost is an issue, or to address side effects by adjusting medications or doses. Communication and coordination with the other health care providers is also a major responsibility of the stroke NPs. For patients with severe stroke deficits, information about medication use is obtained from the caregiver.

The next telephone contact is at 3 months for ascertainment of outcomes including the modified Rankin scale (mRS) (18), Stroke Impact Scale (functional status with the SIS-16 and social domain) (19, 20) primary care visits, change in insurance status, recurrent stroke, or TIA, quality of life (EuroQOL-5-D) (21), Emergency Department visits or hospital readmissions (diagnosis), cigarette smoking, working status, living status, household income adequacy. A major portion of the 3-month outcome interview focuses on a comparison of medications between the stroke discharge and the stroke follow-up clinic, whichever occurred last, in order to assess persistence, as well as reasons for discontinuation. Efforts in TRACS have focused on enrollment and early follow-up. With limited personnel to perform 3-month calls, medication use (persistence) was obtained by review of the Wake Forest Baptist Medical Center (WFBMC) electronic health records (EHR).

### TRACS personnel

The TRACS team includes the TRACS coach and a stroke NP, which allowed access to earlier stroke clinic follow-up than was available with full resident and faculty clinics. Two years after the initiation of the program, we hired a second stroke NP, which then provided the opportunity for continuity of care from the inpatient to the outpatient setting. The stroke NPs, TRACS coach, and TRACS director all worked to develop a pathway for follow-up calls and the timing for clinic visits. The identification of high-risk profiles was based on an analysis of our 30-day readmission case–control study showing that patients with prior hospitalizations before the stroke are at the highest risk for readmission within 30 days (22). The stroke NPs developed the standardized follow-up clinic assessment using a template in the EHR.

## Program Evaluation

By developing a database for entering data about medical history, health literacy, and availability of resources for participants, we created a system that could be queried to evaluate quality improvement efforts. We evaluated patients admitted to WFBMC and discharged home between October 2012 and February 2014. Patients were eligible for TRACS, if they were age 18 years or older and hospitalization for a primary diagnosis of acute ischemic or hemorrhagic stroke, or TIA. Patients who have been discharged to a skilled nursing facility or outside inpatient rehabilitation facility were excluded from the analysis, but are still seen in the NP follow-up clinic. Patients were contacted by the TRACS coach or the stroke NPs according to the schedule above using telephone follow-up or the NP follow-up clinic notes to ascertain stroke prevention medication persistence.

Subjects prescribed an individual medication at discharge, but who were not taking that medication at follow-up (clinic or phone interview), were defined as “non-persistent.” Data collection forms were programed for online data entry of baseline variables using research electronic data capture (REDcap). The study protocol for this analysis was approved by the Wake Forest School of Medicine Institutional Review Board. The coach obtained verbal consent during the enrollment process in the hospital.

### Early program results

Of the 171 patients enrolled, 15 patients (8.8%) were discharged to a skilled nursing facility or outside inpatient rehabilitation facility, and 14 patients (8.2%) were lost to follow-up. A total of 142 patients were included in this analysis. Table 1 shows the baseline characteristics of the analysis cohort. The mean age was 63.6 (range 26–100) years and 52.8% were women, and the proportions with risk factors are shown in Table 1.

**Table 1:** **Baseline characteristics of TRACS patients**.

	Total (*n* = 142)	IS (*n* = 108)	HS (*n* = 13)	TIA (*n* = 21)	*p*-value
**Demographics**				
Age, years	63.6 (13.13)	62.8 (13.43)	62.1 (8.98)	68.8 (12.96)	0.146
Female	75 (52.8)	55 (50.9)	7 (53.8)	13 (61.9)	0.652
**Medical history**				
Hypertension	127 (89.4)	96 (88.9)	12 (92.3)	19 (90.5)	0.918
Diabetes mellitus	51 (35.9)	37 (34.3)	5 (38.5)	9 (42.9)	0.739
Hypercholesterolemia	76 (53.5)	57 (52.8)	5 (38.5)	14 (66.7)	0.263
Coronary artery disease	35 (24.6)	29 (26.9)	2 (15.4)	4 (19.0)	0.539
Prior stroke/TIA	35 (24.6)	25 (23.1)	2 (15.4)	8 (38.1)	0.250
Congestive heart failure	18 (12.7)	12 (11.1)	3 (23.1)	3 (14.3)	0.459
Smoking	40 (28.2)	36 (33.3)	3 (23.1)	1 (4.8)	**0.026**
Alcohol abuse	15 (10.6)	12 (11.1)	1 (7.7)	2 (9.5)	0.918
Depression	18 (12.7)	16 (14.8)	0 (0)	2 (9.5)	0.283
Prior hospitalization	27 (19.0)	17 (15.7)	1 (7.7)	9 (42.9)	**0.008**
**Clinical presentation**				
SBP (mmHg)	158.1 (27.03)	156.2 (25.61)	181.9 (39.78)	153.0 (16.70)	**0.003**
DBP (mmHg)	85.5 (18.43)	85.1 (16.29)	101.1 (30.08)	77.9 (14.61)	**0.001**
HbA1c (%)	6.7 (2.07)	6.8 (2.16)	5.9 (1.29)	6.6 (1.68)	0.655
Total cholesterol (mg/dL)	181.4 (46.08)	182.4 (43.62)	176.1 (44.77)	177.9 (61.06)	0.881
LDL-cholesterol (mg/dL)	109.8 (35.88)	111.9 (34.34)	102.3 (48.65)	100.2 (39.78)	0.397
HDL-cholesterol (mg/dL)	40.0 (12.47)	40.2 (11.97)	34.6 (5.76)	47.3 (15.03)	**0.024**
Triglyceride (mg/dL)	156.0 (119.6)	155.3 (108.35)	212.3 (147.08)	134.9 (162.77)	0.314
**Neurological scale score, median (range)**				
Baseline NIHSS score	2.0 (0–32)	3.0 (0–32)	6.0 (0– 27)	0.0 (0– 9)	**0.004**
mRS at discharge	3.0 (0–5)	3.0 (0–5)	3.0 (1– 4)	0.0 (0– 0)	**0.019**

*IS, ischemic stroke; HS, hemorrhagic stroke; TIA, transient ischemic attack; SBP, systolic blood pressure; DBP, diastolic blood pressure; LDL, low-density lipoprotein; HDL, high-density lipoprotein; NIHSS, National Institutes of Health stroke scale; mRS, modified Rankin score. Bold text used to show variables with significant p values*.

*Data are means (SD) or numbers (%)*.

The overall medication regimen persistence (all medications the same from discharge to follow-up) was 80.3%. The highest persistence was with antithrombotic therapy (96.7%), followed by diabetes medications (95.8%), lipid-lowering (86.7%), and antihypertensive drugs (86.3%). Of the classes of antihypertensive drugs, the lowest persistence was with calcium channel blockers (78.1%), with 9.4% discontinued by the provider and the other 9.4% discontinued by the patient. The factors associated with persistence are shown in Table 2. Persistence was higher in those patients with a first-ever stroke (78.9%) vs. those with recurrent stroke (60.7%; *p* = 0.045), whereas none of the other factors were associated with persistence.

**Table 2:** **Factors associated with medication persistence in TRACS**.

Variable	Persistent (*n* = 114)	Non-persistent (*n* = 28)	*p*-value
Age (years)	63.4 (12.95)	64.29 (14.05)	0.756
Female	59 (51.8)	16 (57.1)	0.609
**Race**		
White	67 (76.1)	21 (23.9)	0.087
African American	23 (79.3)	6 (20.7)	
Not reported	24 (96.0)	1 (4.0)	
**Education level**		
≥college	19 (16.7)	7 (25.0)	0.250
**Work status**		
Working	32 (28.1)	4 (14.3)	0.249
Retired	35 (30.7)	13 (46.4)	
Disabled	10 (8.8)	7 (25.0)	
Not reported	37 (32.4)	4 (14.3)	
**Medical history**		
Hypertension	102 (89.5)	25 (89.3)	0.977
Diabetes mellitus	42 (36.8)	9 (32.1)	0.642
Hypercholesterolemia	60 (52.6)	16 (57.1)	0.668
Coronary artery disease	29 (25.4)	6 (21.4)	0.659
Prior stroke/TIA	23 (21.1)	11 (39.3)	**0.045**
**System factors**		
Have insurance or help to pay for meds	94 (82.5)	22 (78.6)	0.577
Length of admission (days)	3.2 (3.53)	3.7 (2.87)	0.509
Prior hospitalization	24 (21.1)	3 (14.3)	0.212
One or more office visits after discharge	93 (81.6)	22 (78.6)	0.716

*Bold text used to show variables with significant p values*.

Multivariate logistic regression modeling revealed prior stroke/TIA was negatively associated with persistence (OR 0.21, 95% CI 0.06–0.72; *p* = 0.013) after adjustment for age, sex, race, education, insurance, and office visits after discharge. There was also a trend toward prior hospitalization (before the index stroke) and persistence (OR 4.73, 95% CI 0.99–22.50; *p* = 0.051).

## Discussion

The Institute of Medicine recommends integrated approach to evidence-based prevention that also promotes patient access to this care (23). We believe TRACS is an example of a model of stroke prevention and transitional care that meets the Institute of Medicine recommendation because of (1) appropriate initiation of guideline-recommended prevention medications prior to discharge; (2) enhanced literacy-appropriate education provided at discharge with close telephone and clinic follow-up; (3) assessment of medication adherence/persistence; and (4) a triage system to provide community referrals for those patients who have problems with access to medications, regardless of insurance status. It may be difficult to ask patients if they are having financial difficulties, but asking about medication-taking and access to those medications opens a discussion with patients about their specific challenges. If medication non-persistence is not recognized, these patients are at even higher risk for recurrent stroke.

We also showed that analysis of the hospital and outpatient EHR, and coach-acquired data can lead to identification of a subgroup of patients at highest risk for non-persistence – those who have already had a stroke prior to enrollment in TRACS. This was independently associated with non-persistence, even after adjustment for relevant sociodemographic factors. It is possible that non-persistence had been a chronic problem in these patients with recurrent stroke, but perhaps had not been adequately addressed with follow-up after the initial stroke or TIA. The other possibility is that the patients with multiple strokes have risk factors that are the most difficult to control, although we did not find specific risk factors that were associated with non-persistence in our data. For those who are hospitalized prior to a stroke, perhaps this is an interaction that could improve persistence through understanding the patient’s current medication-taking behavior and adjusting medications accordingly. TRACS will be the ideal platform to test new interventions for medication adherence comparing outcomes in those who have a history of a prior stroke vs. those with first-ever stroke.

Patients’ beliefs about medications can have a significant impact on adherence to stroke prevention medications. In Sweden, investigators reported that in nearly 600 stroke survivors, non-adherent participants scored lower on positive beliefs about medications (necessity and benefit) and higher on negative beliefs (concern about harm) and they believed their current treatment to be less useful (24). A synthesis of qualitative studies of medication non-adherence concluded that the main theme was that patients do not take their medications because of a reluctance to take medicines in general, and prefer to take as little as possible (25). Therefore, providers may need to find more effective ways of acknowledging concerns patients have about medications and also highlighting the necessity and benefit of reducing future stroke risk. With the evidence about patient beliefs and concerns, and the assumption that addressing these could lead to improved prevention, researchers in the United Kingdom have designed a pharmacist-led home-based clinical medication review intervention that is currently under investigation in a randomized controlled trial (26).

In summary, we believe that a comprehensive model of stroke prevention should include recognition of non-adherence, and understanding of the factors associated with this phenomenon. The TRACS program is designed with medication-taking behavior at the forefront, and will provide a platform for both quality improvement efforts and design of new interventions to improve adherence. With multiple clinical trials of organizational and behavioral interventions currently underway, additional strategies can be incorporated into the TRACS model to enhance adherence and prevention of recurrent stroke even further.

## Conflict of Interest Statement

The authors declare that the research was conducted in the absence of any commercial or financial relationships that could be construed as a potential conflict of interest.

## References

[1] Go AS Mozaffarian D Roger VL Benjamin EJ Berry JD Blaha MJ Heart disease and stroke statistics – 2014 update a report from the American Heart Association. Circulation (2014) 129:e28–292. 10.1161/01.cir.0000441139.02102.80 24352519 PMC5408159

[2] Kernan WN Ovbiagele B Black HR Bravata DM Chimowitz MI Ezekowitz MD Guidelines for the prevention of stroke in patients with stroke and transient ischemic attack a guideline for healthcare professionals from the American Heart Association/American Stroke Association. Stroke (2014) 45(7):2160–236. 10.1161/STR.0000000000000024 24788967

[3] Heidenreich P. Patient adherence: the next frontier in quality improvement. Am J Med (2004) 117:130–2. 10.1016/j.amjmed.2004.03.007 15234651

[4] Lager KE Mistri AK Khunti K Haunton VJ Sett AK Wilson AD. Interventions for improving modifiable risk factor control in the secondary prevention of stroke. Cochrane Database of Systematic Reviews. John Wiley & Sons, Ltd (2014). Available from: http://onlinelibrary.wiley.com/doi/10.1002/14651858.CD009103.pub2/abstract 10.1002/14651858.CD009103.pub2 24789063

[5] Gehi AK Ali S Na B Whooley MA. Self-reported medication adherence and cardiovascular events in patients with stable coronary heart disease: the heart and soul study. Arch Intern Med (2007) 167:1798–803. 10.1001/archinte.167.16.1798 17846400 PMC2789555

[6] Ovbiagele B Campbell S Faiz A Chambless L. Relationship between non-specific prescription pill adherence and ischemic stroke outcomes. Cerebrovasc Dis (2010) 29:146–53. 10.1159/000262311 19955739 PMC2813814

[7] Bailey J Wan J Tang J Ghani M Cushman W. Antihypertensive medication adherence, ambulatory visits, and risk of stroke and death. J Gen Intern Med (2010) 25:495–503. 10.1007/s11606-009-1240-1 20165989 PMC2869423

[8] Shaya F El Khoury A Mullins C Du D Skolasky R Fatodu H Drug therapy persistence and stroke recurrence. Am J Manag Care (2006) 12:313–9. 16756450

[9] Rodriguez F Cannon CP Steg PG Kumbhani DJ Goto S Smith SC Predictors of long-term adherence to evidence-based cardiovascular disease medications in outpatients with stable atherothrombotic disease: findings from the reach registry. Clin Cardiol (2013) 36:721–7. 10.1002/clc.22217 24166484 PMC6649635

[10] Bushnell C Zimmer L Schwamm L Goldstein L Clapp-Channing N Harding T The adherence evaluation after ischemic stroke longitudinal (AVAIL) registry: design, rationale, and baseline patient characteristics. Am Heart J (2009) 157:428–35. 10.1016/j.ahj.2008.11.002 19249411

[11] Bushnell CD Zimmer LO Pan W Olson DM Zhao X Meteleva T Persistence with stroke prevention medications 3 months after hospitalization. Arch Neurol (2010) 67:1456–63. 10.1001/archneurol.2010.190 20697032 PMC10197116

[12] Bushnell C Olson D Zhao X Pan W Zimmer L Goldstein L Secondary preventive medication persistence and adherence one-year after stroke. Neurology (2011) 77:1182–90. 10.1212/WNL.0b013e31822f0423 21900638 PMC3265047

[13] Lummis H Sketris I Gubitz G Joffres M Flowerdew G. Medication persistence rates and factors associated with persistence in patients following stroke: a cohort study. BMC Neurol (2008) 8:25. 10.1186/1471-2377-8-25 18616796 PMC2474854

[14] Glader E-V Sjolander M Eriksson M Lundberg M. Persistent use of secondary preventive drugs declines rapidly during the first 2 years after stroke. Stroke (2010) 41:2552–8. 10.1161/STROKEAHA.109.566950 20075360

[15] Ovbiagele B Saver JL Fredieu A Suzuki S Selco S Rajajee V In-hospital initiation of secondary stroke prevention therapies yields high rates of adherence at follow-up. Stroke (2004) 35:2879–83. 10.1161/01.STR.0000147967.49567.d6 15514170

[16] Ovbiagele B Kidwell C Selco S Razinia T Saver J. Treatment adherence rates one year after initiation of a systematic hospital-based stroke prevention program. Cerebrovasc Dis (2005) 20:280–2. 10.1159/000087711 16127271

[17] Sides E Zimmer L Wilson L Pan W Olson D Peterson ED Medication coaching program for patients with minor stroke or TIA: a pilot study. BMC Public Health (2012) 12:549. 10.1186/1471-2458-12-549 22830539 PMC3490769

[18] Rankin J. Cerebral vascular accidents in patients over the age of 60: II. Prognosis. Scott Med J (1957) 2:200–15 13432835 10.1177/003693305700200504

[19] Duncan PW Wallace D Lai SM Johnson D Embretson S Laster LJ. The Stroke Impact Scale version 2.0 evaluation of reliability, validity, and sensitivity to change. Stroke (1999) 30:2131–40. 10.1161/01.STR.30.10.2131 10512918

[20] Duncan P Lai S Bode R Perera S DeRosa J. Stroke Impact Scale-16: a brief assessment of physical function. Neurology (2003) 60:291–6. 10.1212/01.WNL.0000041493.65665.D6 12552047

[21] The EuroQol Group. EuroQol* – a new facility for the measurement of health-related quality of life. Health Policy (1990) 16:199–208. 10.1016/0168-8510(90)90421-9 10109801

[22] Strowd RE Wise SM Umesi UN Bishop L Craig J Lefkowitz D Predictors of 30-day hospital readmission following ischemic and hemorrhagic stroke. Am J Med Qual (2014). 10.1177/1062860614535838 24919597

[23] Institute of Medicine. Crossing the Quality Chasm: A New Health System for the 21st Century. Washington, DC: National Academy Press (2001). 25057539

[24] Sjolander M Eriksson M Glader E-L. The association between patients’ beliefs about medicines and adherence to drug treatment after stroke: a cross-sectional questionnaire survey. BMJ Open (2013) 3(9):e003551. 10.1136/bmjopen-2013-003551 24068768 PMC3787491

[25] Pound P Britten N Morgan M Yardley L Pope C Daker-White G Resisting medicines: a synthesis of qualitative studies of medicine taking. Soc Sci Med (2005) 61:133–55. 10.1016/j.socscimed.2004.11.063 15847968

[26] Souter C Kinnear A Kinnear M Mead G. Optimisation of secondary prevention of stroke: a qualitative study of stroke patients’ beliefs, concerns and difficulties with their medicines. Int J Pharm Pract (2014). 10.1111/ijpp.12104 24606322

